# Autochthonous Human Milk *Lactobacillus* Strains as Potential Probiotic Starter Cultures

**DOI:** 10.17113/ftb.64.01.26.9074

**Published:** 2026-02-15

**Authors:** Katarina Butorac, Martina Banić, Dina El Khalifa, Ena Habuš, Nina Čuljak, Andreja Leboš Pavunc, Jasna Novak, Jagoda Šušković, Blaženka Kos

**Affiliations:** Laboratory for Antibiotic, Enzyme, Probiotic and Starter Cultures Technology, University of Zagreb Faculty of Food Technology and Biotechnology, Pierottijeva 6, 10000 Zagreb, Croatia

**Keywords:** human milk, microbiota, functional starter cultures, lactic acid bacteria

## Abstract

**Research background:**

Human milk is rich in bioactive molecules and beneficial bacteria that contribute to shaping the newborn's microbiota. In this study, we aim to evaluate lactic acid bacteria strains isolated from human milk of healthy Croatian women as potential functional starter cultures.

**Experimental approach:**

In order to define novel potential probiotics for use in dairy products, eight strains of lactobacilli were analysed for their proteolytic, antimicrobial and antioxidant activity as well as their survival rate during freeze-drying.

**Results and conclusions:**

Based on the results obtained, the exopolysaccharide-producing *Limosilactobacillus fermentum* MC1, the surface (S)-layer-producing *Levilactobacillus brevis* MB2 and the plantaricin-producing *Lactiplantibacillus plantarum* MB18 strains are candidates for the production of fermented dairy products with potential functional and nutritional relevance for the host. The selected strains exerted high casein degradation capacity, a broad spectrum of antimicrobial activity and a promising 2,2-diphenyl-1-picrylhydrazyl hydrate radical scavenging activity. They also fulfilled the primary technological criterion by having a high survival rate during freeze-drying.

**Novelty and scientific contribution:**

The data presented emphasise the importance of human milk as a valuable source of lactic acid bacteria with unique technological and functional properties, which are important both as a basis for scientific research and for the development of novel starter cultures for functional products.

## INTRODUCTION

Human milk is a source of numerous bioactive molecules that are crucial for the protection and development of the infant. Although it was originally considered sterile, accumulating evidence suggests that it comprises not only biomolecules but also the human milk microbiota, which is of great importance as its disruption, particularly in early childhood, may underlie the development of certain diseases (*1*). Lactic acid bacteria (LAB) with probiotic properties have been isolated from many different sources, but those from human milk are considered valuable because of their human origin and safety for infants (*2*). The most abundant bacterial species in the human milk microbiota belong to the genera *Staphylococcus, Streptococcus, Lactobacillus, Pseudomonas, Corynebacterium, Bifidobacterium, Enterococcus, Rothia, Acinetobacter, Veillonella, Cutibacterium* and *Bacteroides* (*3*, *4*). *Lactobacillus* strains, especially those isolated from fermented milk products, have a highly efficient proteolytic system which ensures self-sufficiency in free amino acids, but is also important for the production of casein-derived bioactive peptides (*5*, *6*). Various LAB strains show antagonistic activity against certain pathogenic microorganisms, which is often attributed to two mechanisms of action (*7*). Firstly, LAB produce organic acids through fermentation, which lowers the pH of the environment and prevents the survival of some pathogens that are intolerable to acidic conditions (*8*). Secondly, LAB can produce bacteriocins, polypeptides that inhibit certain foodborne pathogens and harmful bacteria such as *Escherichia coli*, *Staphylococcus*, *Salmonella* and *Listeria monocytogenes* (*9*, *10*). LAB can also produce surface (S)-layer proteins on their cell surface, which play an important role in the probiotic properties of the producer strain, such as protection against unfavourable environmental conditions, aggregation capacity and adhesion (*11*). Exopolysaccharides (EPSs), high molecular mass carbohydrate polymers, also contribute to the probiotic properties of LAB by favourably influencing their survival during freeze-drying, adherence to human epithelial cells and competitive exclusion of pathogens (*12*).

Numerous health issues such as diabetes, cancer and cardiovascular, neurological and inflammatory disorders are associated with oxidative stress. Probiotics have garnered growing scientific attention due to the ongoing search for natural antioxidants. Although various LAB strains are currently used in numerous dietary supplements, their antioxidant effects are still relatively poorly understood (*13*). For these reasons, LAB with desirable probiotic properties are being used to develop functional beverages that can improve the health of the gastrointestinal tract of consumers by preventing the proliferation of pathogens and activating the immune system (*14*). Some probiotic strains of lactobacilli and bifidobacteria are frequently used as starter and co-starter cultures for the production of various functional products. They tolerate the low pH of products such as fermented milk during fermentation and cold storage (*15*). Therefore, this work focuses on the selection of proteinase-producing, antimicrobial and antioxidant LAB strains previously isolated from the human milk microbiota (*3*) and characterised as potential probiotics (*3*, *12*) to be utilised as functional starter cultures in the production of probiotic products.

## MATERIALS AND METHODS

### Bacterial strains and cultivation conditions

The bacterial strains analysed in this study are deposited in the Culture Collection of the Laboratory for Antibiotic, Enzyme, Probiotic and Starter Cultures Technology, University of Zagreb Faculty of Food Technology and Biotechnology, Zagreb, Croatia. The strains were stored as frozen stocks at -80 °C in a CryoCube F101h ultra-low temperature freezer (Eppendorf, Hamburg, Germany) (Table 1 (*3*, *16*)), in de Man-Rogosa-Sharpe (MRS; Difco, Detroit, MI, USA) broth for lactobacilli, M17 broth (Biolife, Milan, Italy) for lactococci and enterococci and nutrient broth (Biolife) for test microorganisms, supplemented with *φ*(glycerol)=15 % (Sigma-Aldrich, Merck, St Louis, MO, USA). Before every experimental trial, each strain was subcultured twice in a suitable growth medium under the listed growth conditions.

**Table 1:** Bacterial strains used in this study and their cultivation conditions

Bacterial strain	Cultivation conditions	Origin	Bioactive molecule production [reference]
*Lactiplantibacillus* *plantarum* KR19	MRS, 37 °C, microaerophilic*	human milk	bacteriocin (*3*)
*Limosilactobacillus fermentum* MC1	MRS, 37 °C, microaerophilic*	human milk	exopolysaccharide (*3*)
*Lactiplantibacillus* *plantarum* MC19	MRS, 37 °C, microaerophilic*	human milk	bacteriocin (*3*)
*Levilactobacillus* *brevis* MB1	MRS, 37 °C, microaerophilic*	human milk	S-protein (*3*)
*Levilactobacillus* *brevis* MB2	MRS, 37 °C, microaerophilic*	human milk	S-protein (*3*)
*Levilactobacillus* *brevis* MB13	MRS, 37 °C, microaerophilic*	human milk	S-protein (*3*)
*Lactiplantibacillus* *plantarum* MB18	MRS, 37 °C, microaerophilic*	human milk	bacteriocin (*3*)
*Levilactobacillus* *brevis* MB20	MRS, 37 °C, microaerophilic*	human milk	S-protein (*3*)
*Lactobacillus* *helveticus* M92	MRS, 37 °C, microaerophilic*	fermented milk	S-protein (*16*)
*Lactococcus lactis* ssp. *lactis* LMG 9450	M17, 30 °C, aerobic	BCCM	/
*Enterococcus faecium* ATCC^®^9430^™^	M17, 37 °C, aerobic	ATCC	/
*Staphylococcus aureus* ATCC^®^25925^™^	nutrient broth, 37 °C, aerobic	ATCC	/
*Listeria monocytogenes* ATCC^®^19111^™^	nutrient broth, 37 °C, aerobic	ATCC	/
*Escherichia coli* ATCC^®^25922^™^	nutrient broth, 37 °C, aerobic	ATCC	/
*Salmonella enterica* serovar Typhimurium ATCC^®^14028^™^	nutrient broth, 37 °C, aerobic	ATCC	/

*Microaerophilic conditions were created using Anaerocult A system (Merck, Darmstadt, Germany). BCCM=Belgian Coordinated Collections of Microorganisms, ATCC=American Type Culture Collection

### Antimicrobial activity

The antimicrobial activity of the LAB strains against test microorganisms and related LAB was determined using the turbidimetric method according to Leboš Pavunc *et al*. (*17*) with slight modifications. The supernatant of overnight cultures of LAB strains isolated from human milk was filtered using sterile filter (Sigma-Aldrich, Merck) with a diameter of 0.2 μm, and the filtrate was used in the experiment.

The antimicrobial activity of the selected LAB isolates from human milk was tested against test microorganisms (*Staphylococcus aureus* ATCC^®^25925^™^, *Listeria monocytogenes* ATCC^®^19111^™^, *Escherichia coli* ATCC^®^25922^™^ and *Salmonella* Typhimurium FP1) and related LAB strains (*Lactobacillus helveticus* M92, *L. lactis* ssp. *lactis* LMG 9450 and *Enterococcus faecium* ATCC^®^9430^™^) cultured in the appropriate medium until their *A*_620 nm_ reached 0.5 and 1.0, respectively. A volume of 10 µL of overnight cultures of the test microorganisms or related LAB strains was added to the wells of the microtitre plate (Greiner Bio-One, Kremsmünster, Austria) together with 90 µL of a suitable cultivation medium and 100 µL of culture supernatants of selected LAB isolates from human milk. A volume of 10 µL of a culture of a specific test microorganism or a related LAB grown in 190 µL of the suitable cultivation medium was used as a control. The antibacterial activity of the culture supernatant was determined after 24 h at 37 °C by spectrophotometric measurement of the apparent absorbance at a wavelength of 620 nm using a microtitre plate reader Infinite F Plex (Tecan, Männedorf, Switzerland).

### Proteolytic activity

#### Determination of fast milk coagulation phenotype and acidification capacity

A volume of 5 mL of the overnight cultures of the LAB strains was centrifuged using an Eppendorf 5804 R centrifuge (Eppendorf, Hamburg, Germany) at 8000×*g* for 10 min at 4 °C. The cells were washed twice with sterile phosphate-buffered saline (PBS, pH=7.4). A volume of 200 µL of each cell suspension was inoculated into 10 % (*m*/*V*) skimmed milk (Sigma-Aldrich, Merck) and incubated at 37 °C for 16 h.

The ability of the LAB to coagulate milk was determined according to Hebert *et al*. (*18*). Depending on the rate of milk coagulation, the results were interpreted as follows: no coagulation, or as low, moderate, good or excellent coagulation.

Acidification capacity was measured in the supernatants by monitoring the pH change with a pH meter (Metrohm, Herisau, Switzerland) and by the titration method with 0.1 M NaOH (Carlo Erba, Milan, Italy) with the addition of phenolphthalein indicator (Kemika, Zagreb, Croatia) until a pink colour appeared. The titratable acidity expressed as °SH (1 °SH=0.0225 % lactic acid) was determined as follows:


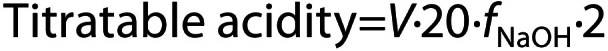
 /1/

where *V* is the volume (in mL) of 0.1 M NaOH and *f*_NaOH_ is the correction factor of NaOH (1).

#### Proteinase plate assay

Mass per volume ratio of 10 % skimmed milk (Sigma-Aldrich, Merck) was used to observe the proteinase phenotype of the strains according to Raveschot *et al*. (*19*) with slight modifications. Solid skimmed milk agar plates were routinely prepared by adding 1 % (*m*/*V*) agar (Biolife) to the medium. After solidification, sterile wells with a diameter of 7 mm were drilled and 50 µL of cell suspension or the supernatant of the overnight grown bacterial culture was added. Transparent halos were an indicator of proteolytic activity.

#### Determination of proteolytic activity by Anson's method

The proteolytic activity of selected LAB strains was determined using the Anson method according to Beganović *et al*. (*16*). A volume of 1 mL of the supernatant filtrate of the overnight culture of each strain was suspended with 5 mL of a 0.65 % (*m*/*V*) casein solution in PBS (pH=7.2). After a 10-minute incubation at 37 °C, the reaction was halted by adding 5 mL of trichloroacetic acid (Thermo Fischer Scientific, Waltham, MA, USA), which led to precipitation of the non-hydrolysed proteins. After another incubation (30 min, 37 °C) and filtration, 5 mL of 0.4 M Na_2_CO_3_ solution (Kemika, Zagreb, Croatia) and 1 mL of Folin-Ciocalteu phenol reagent (Merck, Darmstadt, Germany) were added to 2 mL of filtrate. The sample was incubated again for 30 min at 37 °C and filtered. The absorbance was measured at 670 nm on an LKB 5060-006 microplate reader (LKB Vertriebs GmbH, Vienna, Austria). The blank contained a pre-incubated casein solution to which trichloroacetic acid was added at the beginning of the experiment, followed by the addition of the supernatant and all the procedures described above.

**Fig. 1:**
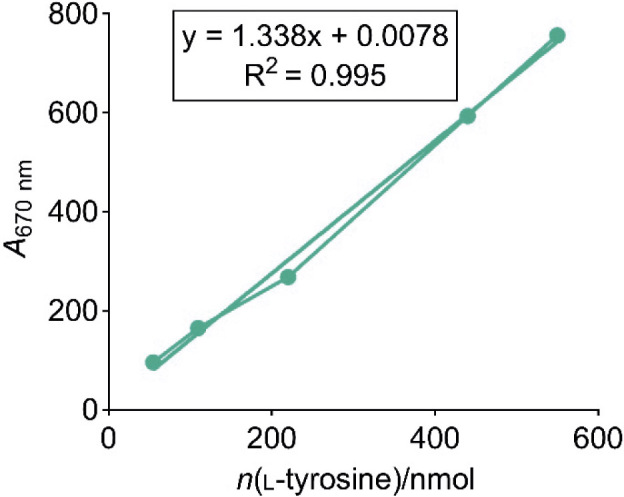
Standard curve for the measurement of amount of l-tyrosine and corresponding linear equation for the determination of proteolytic activity by Anson's method

Based on the linear equation, the amount (nmol) of l-tyrosine (Merck) released was calculated by measuring the absorbance (*A*_670 nm_), resulting from the hydrolysis of casein by proteases in the samples (Fig. 1).

#### Analysis of the casein degradation products by Tris-tricine SDS-PAGE

The caseionolytic activity of selected bacterial strains was tested according to El-Ghaish *et al*. (*20*) with modifications. Overnight cultures were centrifuged using an Eppendorf 5804 R centrifuge at 3600×*g* for 10 min and then washed with phosphate buffer (pH=7.4). The resulting biomass was suspended in a 2 % (*m*/*V*) skimmed milk solution (Sigma-Aldrich, Merck) and incubated at 37 °C for 48 h. Casein degradation was then monitored using the Tris-tricine sodium dodecyl sulphate-polyacrylamide gel electrophoresis (SDS-PAGE) method. A non-inoculated skimmed milk solution was used as a control. The gel for Tris-tricine SDS-PAGE was prepared according to Haider *et al*. (*21*).

### Antioxidant activity

Overnight cultures of LAB strains were washed twice in PBS buffer (pH=7.4), after which the cells were mixed with freshly prepared 2,2-diphenyl-1-picrylhydrazyl hydrate (DPPH) (0.2 mM in ethyl alcohol) in a 1:1 ratio and incubated in the dark for 30 min. After incubation, the samples were centrifuged and the absorbance of the supernatant was measured at 517 nm. Ethanol and PBS buffer (pH=7.4) were used as a blank test, and DPPH solution in ethyl alcohol and PBS buffer (pH=7.4) was used as a control. The ability to remove DPPH radicals was calculated according to the following equation (*22*):


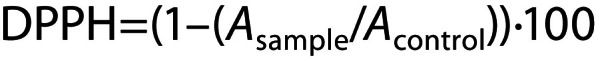
 /2/

where DPPH is the percentage of 2,2-diphenyl-1-picrylhydrazyl hydrate, *A*_sample_ is the absorbance of the sample at 517 nm and *A*_control_ is the absorbance of the control at 517 nm.

### Freeze-drying

Bacterial cells grown to late exponential phase in the optimal liquid nutrient medium were collected by centrifugation using an Eppendorf 5804 R centrifuge (3600×*g*), washed twice with sterile physiological solution and suspended in phosphate buffer (pH=7). The prepared suspension cells were then frozen overnight at -80 °C and freeze-dried for 24 h in Martin Christ Alpha 1–2 LDplus freeze dryer (Osterode, Germany).

### Graphical representation and statistical analysis

The experiments were conducted in triplicate and the results are given as the mean of three independent experiments±standard deviation. All graphs, calculations and statistical analyses were made using GraphPad Prism v. 10.1.1 for Windows (*23*). Ordinary one-way analysis of variance (ANOVA) was performed to calculate the significance of differences among multiple pairs of means in the data group. Differences between groups were considered significant when the p-value was below 0.05.

## RESULTS AND DISCUSSION

### Functional probiotic properties

#### Antibacterial activity

**Fig. 2:**
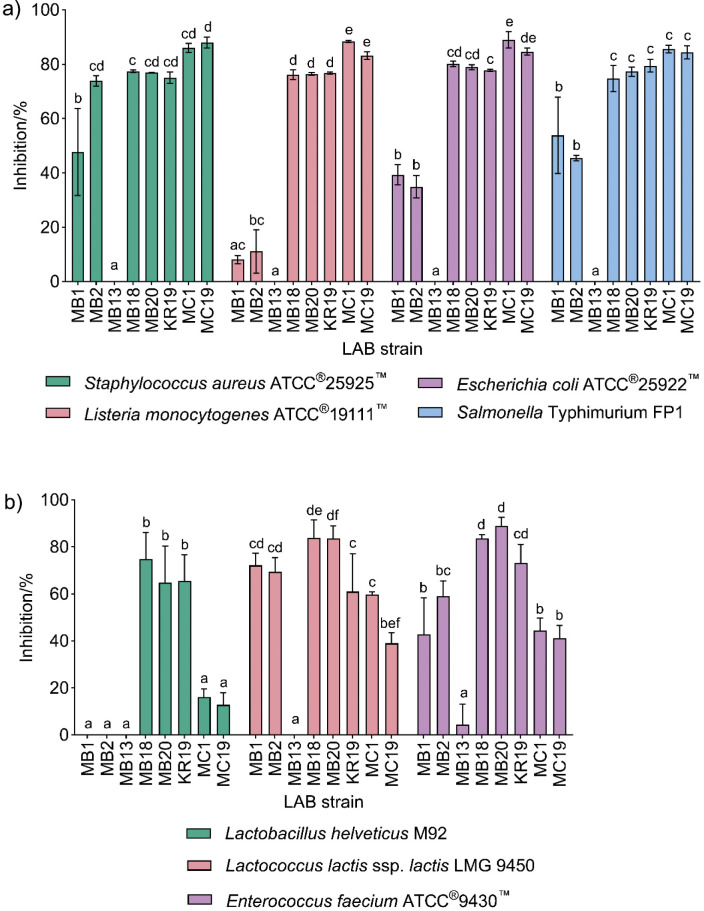
Antimicrobial activity of lactic acid bacteria (LAB) strains against: a) test microorganisms, and b) related LAB strains tested by the turbidimetric method. Different letters above bars indicate statistically significant difference (p<0.05) between the LAB strains and the same test microorganism, *i.e.* a related LAB strain

Isolation of LAB with desired functional properties from human sources is a tantalising approach to select potent probiotics for health-promoting applications (*24*). Selected LAB strains isolated from the human milk of Croatian women were previously identified by 16S RNA and whole genome sequencing, deposited in the NCBI database and characterised as producers of potential biotherapeutic molecules such as S-layer proteins (*L. brevis* MB1, MB2, MB13 and MB20), exopolysaccharides (*L. fermentum* MC1) and plantaricins (*L. plantarum* KR19, MC19 and MB18) (*3*). These strains showed good survival under simulated gastrointestinal tract conditions, aggregation and adhesion to various epithelial and subepithelial structures of the intestinal tract (*11*). The aim of this study was therefore to assess the antimicrobial activity of selected LAB strains using the turbidimetric method in an appropriate liquid growth medium. The results were expressed as growth inhibition (%) of the tested bacteria compared to the control growth. The turbidimetric method was chosen due to the higher sensitivity than agar methods and due to the potential application of selected LAB strains isolated from human milk as functional starter cultures or even probiotics in fermented functional products. According to the results, after 24 h of incubation, the strains *L. plantarum* MC19 and *L. fermentum* MC1 showed the strongest (p<0.05) antimicrobial activity against the test microorganisms, while the antagonistic activity against the related LAB strains was depleted. *L. plantarum* MC19 strongly inhibited *S. aureus* ATCC^®^25925^™^, *S.* Typhimurium ATCC^®^14028^™^, *L. monocytogenes* ATCC^®^19111^™^ and *E. coli* ATCC^®^25922^™^ with inhibition rates of more than 80 %. A similar antimicrobial activity was observed for *L. fermentum* MC1, with a significantly higher inhibition against *L. monocytogenes* ATCC^®^19111^™^ (p=0.004) than strain MC19 (Fig. 2a). A slightly lower, but still very high antimicrobial activity was observed for the strains *L. plantarum* MB18 and KR19 against both the test microorganisms and related LAB strains (Fig. 2b). In contrast, *L. brevis* MB13 showed no activity, while other S-layer expressing strains (*L. brevis* MB1, MB2 and MB20) showed lower activity than other strains tested. The antimicrobial effect of LAB against common foodborne pathogens such as *E*. coli, *L. monocytogenes* and *Salmonella* spp. results from the lowering of the pH of the medium due to the organic acids formed and the activity of synthesised bacteriocins, vitamins, EPSs and other metabolites with proven antimicrobial activity (*25*, *26*). While bacteriocins directly kill competing, closely related bacteria or pathogens or inhibit their growth by various mechanisms such as forming pores in the target cell membrane, disrupting ion gradients or inhibiting cell wall synthesis, S-layer proteins form a crystalline layer that covers the surface of the producing bacteria and contributes to structural integrity, mediates interactions with host tissue and modulates host immune responses (*11*). As a result, S-layers significantly enhance the adhesion of the producer to host cells and prevent pathogen adhesion without having direct bactericidal or bacteriostatic properties. This is probably the reason why S-layer producers have a less potent antimicrobial effect than bacteriocin producers. The *pln* loci of three investigated *L. plantarum* strains were disclosed by the detection of *pln*EF, *pln*A and *pln*J genes responsible for the production of the bacteriocins *Pln*EF and *Pln*A, and the peptide *Pln*J of the plantaricin *Pln*JK, using gene-specific primers (*3*). On the other hand, EPSs have health-promoting and rheological properties in the food, pharmaceutical and nutraceutical industries by exerting antimicrobial, antioxidant, immunomodulatory and many other biological functions. Strain *L. fermentum* MC1 biosynthesises a mixture of three different polymers and harbours the genes involved in EPS production and transport, as well as a gene cluster related to bacteriocin production (*12*), which is a functional property contributing to antimicrobial activity. Strain MC1 also showed excellent adhesion properties, which are important for its probiotic activity. It can therefore be surmised that the strong antimicrobial activity of the plantaricin-producing *L. plantarum* strains and the EPS-producing *L. fermentum* MC1 is due to the biomolecules they produce.

#### Screening of proteolytic activity

**Fig. 3:**
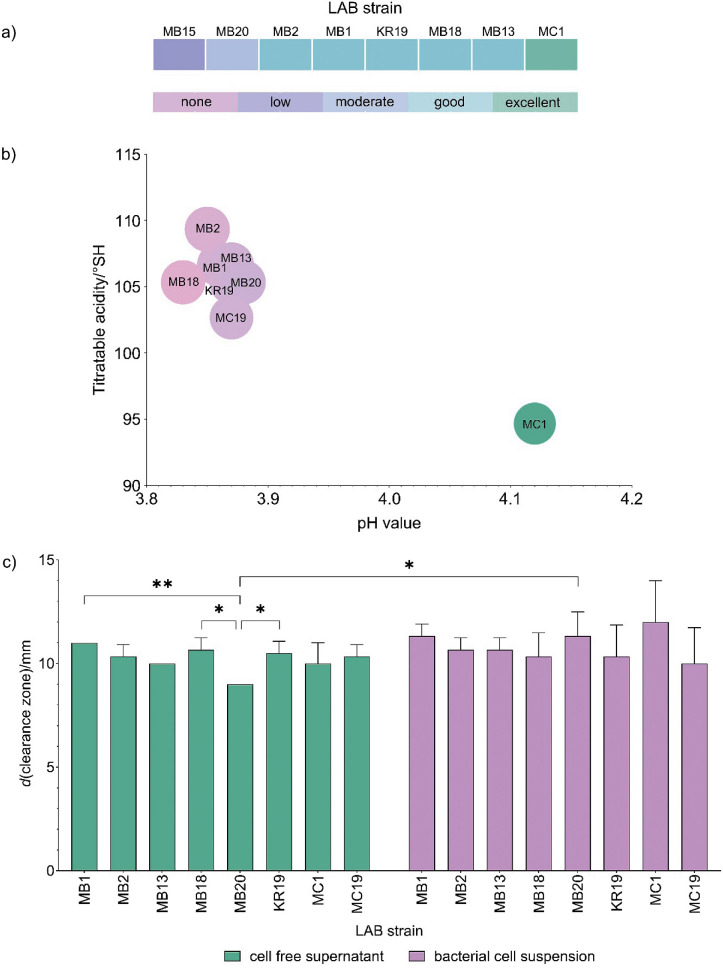
Results of measurements of: a) milk coagulation efficiency, b) pH values and degree of acidity after overnight incubation, and c) proteinase activity of cell-free culture supernatants and concentrated bacterial cell suspensions of *Lactobacillus* strains isolated from human milk. Asterisks indicate significant differences at different levels: *p<0.05, **p<0.01

The main component of human and bovine milk is β-casein, a protein that is a rich source of bioactive and antimicrobial peptides contributing to the endogenous peptidome of milk (*6*, *27*). Therefore, bovine skimmed milk was used as a milk model system for the evaluation of proteolytic capacity. Preliminary analyses of the selected autochthonous strains from human milk have shown that their ability to efficiently coagulate milk and exhibit a fast milk coagulation (Fmc) phenotype is a strain-dependent trait (Fig. 3a). *L. plantarum* MB15 showed a low coagulation efficiency, *L. brevis* MB20 a moderate one, the strains *L. plantarum* KR19, *L. plantarum* MB18, *L. brevis* MB13, *L. brevis* MB1 and *L. brevis* MB2 a good one, while the strain *L. fermentum* MC1 showed an excellent coagulation efficiency. Determination of the fast milk acidification rate showed that the strains decreased the pH of the cultivation medium after overnight growth to values between (4.12±0.01) for *L. fermentum* MC1 and (3.81±0.08) for *L. plantarum* MB18, which is consistent with the degree of acidity (Fig. 3b). Cervantes-Elizarrarás *et al*. (*28*) have reported that organic acids generated by LAB can suppress the growth of Gram-negative bacteria by penetrating their cell membranes and thus impairing their function, leading to acidification of the cytoplasm and inhibition of acid-sensitive enzymes. Although *L. fermentum* MC1 decreased the pH of the medium the least, it exhibited the strongest Fmc+ phenotype as determined by the fastest coagulation rate of the milk. This discrepancy can be explained by the fact that rapid milk coagulation does not necessarily require high acid production, as LAB can possess various traits and mechanisms, such as high enzymatic activity, which allow them to effectively coagulate milk proteins independently of acid production. Presumably, strain MC1 produces proteolytic enzymes that directly cleave milk proteins and cause effective coagulation without significantly lowering the pH of the medium.

Cell-envelope proteinases (CEPs) play a crucial role in the proteolytic system of LAB, as they are required to degrade proteins into peptides and/or amino acids, serving as a nitrogen source for LAB. Therefore, the presence of potential proteinase activity was tested in concentrated bacterial cell suspensions and in cell-free culture supernatants. The result showed that both the strains and their cell-free supernatants were able to hydrolyse the skimmed milk proteins as evident from the appearance of the transparent halos around the wells in the agar, indicating casein hydrolysis (Fig. 3c). The mean diameter of the transparent halos was 10.8±1.2 for the LAB cell suspensions and 10.2±0.7 for the cell-free supernatants, indicating a slightly stronger caseinolytic activity of the LAB cells, albeit without significant meaning (p>0.05). However, the cell suspension of strain *L. brevis* MB20 showed significantly stronger (p=0.036) caseinolytic activity than its cell-free supernatant, suggesting that its proteolytic activity may be due to CEPs. These results are consistent with the research of Novak *et al*. (*6*), in which caseinolytic activity was detected in concentrated cell biomass of lactobacilli and lactococci strains isolated from various autochthonous fermented products.

**Fig. 4:**
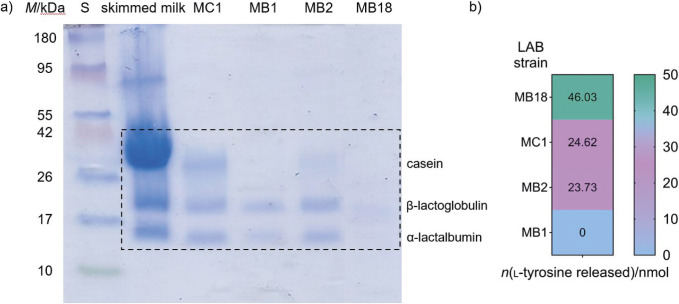
Results of: a) Tris-tricine SDS-PAGE analysis of samples obtained after hydrolysis of skim milk and b) amount (nmol) of l-tyrosine released by potential proteolytic activities of selected lactic acid bacteria (LAB) strains determined by the Anson's method

Representative exopolysaccharide (*L. fermentum* MC1), bacteriocin (*L. plantarum* MB18) and S-protein (*L. brevis* MB1 and *L. brevis* MB2) producers, whose cell-free culture supernatants and concentrated bacterial cell suspensions showed the highest proteinase activity, were further selected for evaluation of casein degradation potential. Human milk contains two classes of proteins, casein and whey proteins (*29*). In this study, the hydrolysis of casein and whey proteins into protein fragments and peptides was studied using Tris-tricine SDS-PAGE (Fig. 4a). This resulted in the appearance of lower-intensity bands, implying that casein and whey proteins were partially degraded, *i.e.* a smaller amount of intact proteins remained. The strain *L. plantarum* MB18 showed the highest proteinase activity, which is consistent with the quantitative analysis of proteinase activities, where the amount of l-tyrosine released was (46.0±8.8) nmoL (Fig. 4b). According to the literature, many LAB belonging to the *L. plantarum* strains produce peptides with numerous bioactive effects such as anti-inflammatory, antihaemolytic, antioxidant, antimutagenic or antimicrobial effects through the fermentation of milk (*30*). Since infant formulas are highly rich in casein, which makes them difficult to digest compared to human milk, supplementation with strains expressing active proteinase could eventually contribute to improved casein digestibility (*31*). This feature is attractive from various aspects of the application of *Lactobacillus* strains, whether as a probiotic supplement in cow's milk-based infant formula or as a starter culture in fermented food products as this can lead to the accumulation of health-promoting bioactive peptides.

#### Antioxidant capacity

An imbalance in the body can be caused by oxidative stress, which leads to damage to cells and tissues, triggered by the excessive production of reactive oxygen species (ROS) and reactive nitrogen species (RNS). As a result, various diseases can develop, such as diabetes, cancer, cardiovascular problems and inflammatory and neurological diseases. For this reason, it is necessary to develop supplements with antioxidant effect in order to reduce oxidative stress, and here the potential of probiotics has also gained tremendous scientific importance (*13*, *32*). During food fermentation, the antioxidant activity of LAB can be attributed to bioactive peptides, EPSs, organic acids and a change in the pH of the environment, which can lead to an increase in their bioavailability (*5*, *33*).

**Fig. 5:**
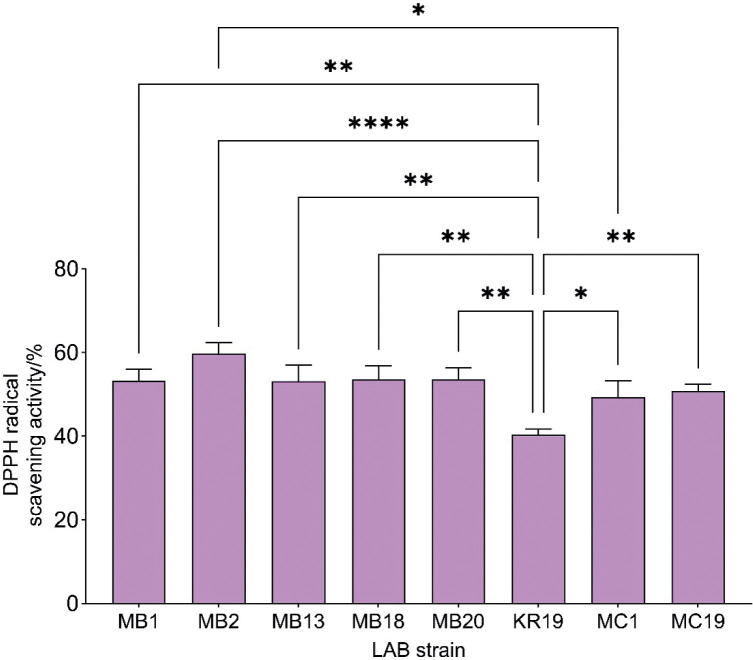
Antioxidant activity as percentage of DPPH scavenging of selected *Lactobacillus* strains. Asterisks indicate significant differences at different levels: *p<0.05, **p<0.01, ****p<0.0001

Therefore, the antioxidant activity of the selected strains was evaluated using DPPH radical scavenging activity. Strain *L. brevis* MB2 showed the highest and strain *L. plantarum* KR19 the lowest radical scavenging activity, while strains MB1, MB13, MB20, MB18, MC1 and MC19 showed similar DPPH radical scavenging activity of about 50 % (Fig. 5). Using the same method, Vougiouklaki *et al*. (*34*) reported that *Lactobacillus gasseri* ATCC 33323 removed 78 % of DPPH radicals, while the values for *Lacticaseibacillus rhamnosus* GG ATCC 53103, *Levilactobacillus brevis* ATCC 8287 and *Lactiplantibacillus plantarum* ATCC 14917 were between 33 and 39 %.

Among all the strains tested, the EPS-producing *L. fermentum* MC1 and the plantaricin-producing *L. plantarum* MB18 strains can find potential application due to their desirable properties. Proteolytic activity is a prerequisite for starter culture application, which can result in production of bioactive peptides with numerous functional properties (*5*). Due to their ability to inhibit the growth of a wide spectrum of bacteria, they can serve as biopreservatives that can extend the shelf life of the consumed product. In addition to antimicrobial properties, antioxidant activity may also be important in extending the shelf life of functional products as well as for protection from oxidative damage in the human body after consumption, along with their functional role, while EPS production can have a positive impact on rheological properties. Overall, all these effects can additionally improve the nutritional relevance of functional products with functional roles for host health, such as gut microbiota balance.

### Technological probiotic properties

Drying processes are often used to stabilise probiotic ingredients by reducing their moisture content and facilitating their transport and preservation, with freeze-drying being the most commonly used method. The probiotic powder acquired by freeze-drying can successfully maintain the viability of probiotics and has a satisfactory fermentation performance (*35*). Although cryoprotectants are often used to support the survivability of bacteria, we used only phosphate buffer to investigate the ability of LAB strains isolated from human milk to survive the extreme conditions during freeze-drying. According to the results (Table 2), the most remarkable survival rate after freeze-drying was observed in *L. brevis* MB1 and MB20 strains, with a loss of only (1.09±0.02) and (1.00±0.05) log CFU/mL, respectively. On the other hand, *L. plantarum* strains showed a greater loss of viable cells, especially strain *L. plantarum* MC19 with a loss of (4.5±0.1) log CFU/mL, while *L. plantarum* MB18 lost only (1.85±0.01) log CFU/mL. This phenomenon may be due to the expression of S-layer proteins on the cell surface of the *L. brevis* strains, which also showed a protective role in simulated gastrointestinal passage and increased adhesion to the Caco-2 cell line (*10*). All strains, with the exception of *L. plantarum* MC19, excreted more than 10^6^ CFU/mL after freeze-drying, which is a generally recognised requirement for probiotics to have a therapeutic effect at the time of consumption (*36*). Overall, all tested strains, with the exception of *L. plantarum* MC19, fulfil the primary technological criterion for selecting probiotic strains.

**Table 2:** The viable cell count of lactic acid bacteria (LAB) strains isolated from human milk before and after freeze-drying in phosphate buffer

LAB strain	Before freeze-drying *N*/(logCFU/mL)	After freeze-drying *N*/(log CFU/mL)	Freeze-drying survival rate/%
*L. plantarum* KR19	(9.722±0.003)^a^	(6.95±0.03)^b^	(71.50±0.03)
*L. fermentum* MC1	(9.96±0.07)^a^	(7.85±0.08)^b^	(78.8±0.1)
*L. plantarum* MC19	(9.66±0.02)^a^	(5.1±0.11)^b^	(53.0±0.1)
*L. brevis* MB1	(9.245±0.004)^a^	(8.16±0.02)^b^	(88.25±0.02)
*L. brevis* MB2	(10.228±0.003)^a^	(8.27±0.02)^b^	(80.88±0.02)
*L. brevis* MB13	(9.67±0.02)^a^	(7.995±0.069)^b^	(82.67±0.07)
*L. plantarum* MB18	(8.86±0.01)^a^	(7.003±0.005)^b^	(79.08±0.01)
*L. brevis* MB20	(9.02±0.02)^a^	(8.01±0.04)^b^	(88.88±0.05)

Results are reported as mean value±standard deviation. Different letters in superscript indicate statistically significant difference (p<0.05) between the same strain before and after freeze-drying

## CONCLUSIONS

Our data provide interesting insights into the specific probiotic features and potential use of LAB isolated from the human milk microbiota in functional products, especially the exopolysaccharide-producing strain *Limosilactobacillus fermentum* MC1 and the plantaricin-producing *Lactiplantibacillus plantarum* MB18. The beneficial properties of these cultures, exerted through functional, technological and safety criteria, may be useful for the production of fermented products with added functional value and with potential nutritional and functional relevance for the host. Their potential application may focus on their use as bio-preservatives to reduce the use of chemical additives, which meets consumer demand for more natural and environmentally friendly products.
